# Adult Neural Stem Cells: Born to Last

**DOI:** 10.3389/fcell.2019.00096

**Published:** 2019-06-04

**Authors:** Aixa V. Morales, Helena Mira

**Affiliations:** ^1^Instituto Cajal, Consejo Superior de Investigaciones Científicas, Madrid, Spain; ^2^Instituto de Biomedicina de Valencia, Consejo Superior de Investigaciones Científicas, Valencia, Spain

**Keywords:** neurogenesis, quiescence, hippocampus, neural stem cell, transcriptional profile

## Abstract

The generation of new neurons is a lifelong process in many vertebrate species that provides an extra level of plasticity to several brain circuits. Frequently, neurogenesis in the adult brain is considered a continuation of earlier developmental processes as it relies in the persistence of neural stem cells, similar to radial glia, known as radial glia-like cells (RGLs). However, adult RGLs are not just leftovers of progenitors that remain in hidden niches in the brain after development has finished. Rather, they seem to be specified and set aside at specific times and places during embryonic and postnatal development. The adult RGLs present several cellular and molecular properties that differ from those observed in developmental radial glial cells such as an extended cell cycle length, acquisition of a quiescence state, a more restricted multipotency and distinct transcriptomic programs underlying those cellular processes. In this minireview, we will discuss the recent attempts to determine how, when and where are the adult RGLs specified.

## Introduction

During the formation of the central nervous system, RGCs proliferate and differentiate to first generate neurons in a process known as neurogenesis and later, in a second wave, glial cells. While the latter process of gliogenesis continues at postnatal stages and it is widespread throughout the adult vertebrate brain (Rowitch and Kriegstein, 2010; Gallo and Deneen, 2014), neurogenesis ceases soon after birth in most mammalian brain regions. In rodents, two exceptions are the SGZ of the DG in the hippocampus and the V-SVZ of the lateral ventricles, in which, respectively, GN and progenitors of olfactory bulb interneurons are generated throughout life (Altman and Das, 1965; Doetsch et al., 1999; Fuentealba et al., 2012; Gonçalves et al., 2016). Adult neurogenesis depends on the persistence of neural stem cells that share properties with developmental RGCs, to which we will refer throughout the review as radial glia-like cells (RGLs). In the adult human brain, RGLs from the V-SVZ are thought to contribute new interneurons to the striatum (Ernst et al., 2014) while the SGZ contributes cells to the DG (Eriksson et al., 1998; Spalding et al., 2013; Boldrini et al., 2018; Moreno-Jiménez et al., 2019). However, adult neurogenesis in humans is still a matter of controversy (Cipriani et al., 2018; Sorrells et al., 2018) and although several technical issues have been considerably improved (Moreno-Jiménez et al., 2019), additional approaches should be undertaken before the whole scientific community accepts its existence (discussed in Lee and Thuret, 2018; Kempermann et al., 2018; Paredes et al., 2018; Snyder, 2018).

The question then arises as to why, at least in most mammals, are the adult neurogenic niches so restricted? And, why is the neurogenic process extended in time and reduced in number in the adult brain? Is it related to specific properties of adult RGLs such as quiescence? When and how is the quiescent pool of RGLs established? This minireview will revisit these questions with a focus on the DG niche of the rodent brain. Nevertheless, some aspects related to V-SVZ neurogenesis will be mentioned.

## The Development of the Dentate Gyrus

One of the approaches to start understanding the uniqueness of the adult neurogenic process is to look at its origin during brain development. The development of the DG is quite distinct, first because it is more protracted in time than that of other cortical regions and also because, in comparison with the neocortex and the rest of the hippocampus, it involves the migration of a separate group of neural progenitors from the neuroepithelium, away from the VZ and close to the pial surface.

**FIGURE 1:**
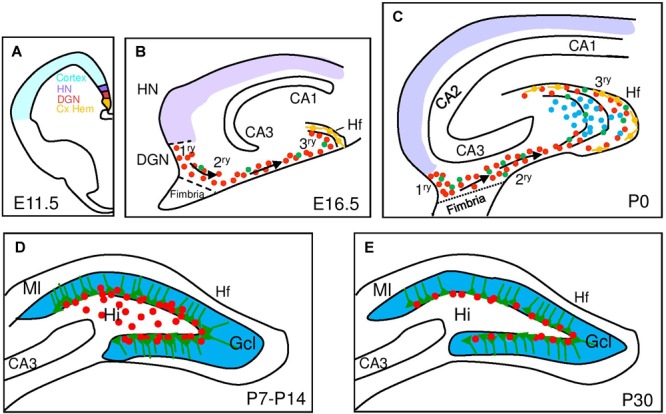
Development of the mouse dentate gyrus. Schematic representation of hippocampal development from embryonic (E) to postnatal stages (P) with a focus on dentate gyrus development. **(A)** At E11.5 signals from the cortical hem (Cx HEM, orange) specify the adjacent dentate gyrus neuroepithelium (DGN, red) and the hippocampus neuroepithelium (HN, purple). **(B)** Late embryonic DG development (E16.5) showing dentate progenitor cells delaminating from the DGN forming the primary matrix (1^ry^) and the migratory stream of proliferating migratory RGCs (green) and progenitor cells (red) forming the secondary matrix (2^ry^) and then the tertiary matrix (3^ry^), formed around the hippocampal fissure (Hf). Cajal-Retzius cells (orange) are derived from the Cx HEM and follow the Hf. **(C)** After birth, the upper and lower blades of the DG are observed. Primary and secondary matrices soon disappear but progenitors in the tertiary matrix continue actively dividing and producing granule neurons with continued insertion of radial glial fibers to organize the addition of new DG neurons. **(D)** At P7 mitotic cells gather at the border between the hilus (Hi) and the granular cell layer (Gcl) and establish the future subgranular zone (SGZ) at P14. **(E)** At P30, mitotically active RGL and IPCs are restricted to the SGZ. CA, Cornu Ammonis; Ml, molecular layer.

The DG progenitors originate at around embryonic day (E) 13.5 in mice from a restricted area of the medial pallium neuroepithelium, the DG neuroepithelium (DGN) or primary (1^ry^) matrix (Altman and Bayer, 1990) that receives patterning signals from the adjacent cortical hem (a hippocampal organizer; Mangale et al., 2008; Figure 1). DG progenitors migrate through the secondary (2^ry^) matrix, next to the fimbria border and toward the pial side of the cortex, forming the dentate migratory stream, composed of a mix of IPCs and postmitotic immature GNs, the principal neuron of the DG. At the end of their migration, GNs accumulate in the DG anlage or hilus and a new germinative pool, called the tertiary (3^ry^) matrix, is established (Figure 1). While DG morphogenesis starts early in embryonic development, the vast majority of GNs are generated within the first two postnatal weeks and originate from the 3^ry^ matrix (Muramatsu et al., 2007). Significantly, between postnatal day (P) 20 and P30, proliferating cells become gradually confined to the SGZ, which serves as source of newly born neurons in the adult DG (Altman and Das, 1965; Altman and Bayer, 1990; Urban and Guillemot, 2014). Thus, the DGN only generates one type of neuron, the GN, and even DG astrocytes will be generated from a different region, the fimbria neuroepithelium, that is a derivative of the cortical hem (Altman and Bayer, 1990).

The majority of adult RGLs emerge in the DG during the first postnatal week. Ablation of proliferating Nestin-creERT^+^ stem cells in this period leads to a lasting depletion of the adult RGL pool in the DG with a corresponding inhibition of adult neurogenesis and a RGL fate bias toward astrocytic progeny (Youssef et al., 2018). However, the same experiment performed from P14-P21 does not alter the pool of adult RGLs and only leads to reduced adult neurogenesis. In the same direction, using a reporter line (Hopx-creERT2) that labels mostly DG neural progenitors during development (Li et al., 2015), it has been genetically determined that a common neural precursor population with a restricted cell lineage continuously and exclusively contributes GNs to the DG formation from the 1^ry^ matrix up to adulthood (Berg et al., 2019). These experiments also confirm that the first progenitors with a typical RGL morphology appear around P7-P8 (Figure 1).

Interestingly, it has been also proposed that a subpopulation of RGLs and neural progenitors along the hippocampal longitudinal axis (septal/dorsal to temporal/ventral axis) is generated in the ventral part of the hippocampus and migrate perinatally from temporal to septal poles before settling (Li et al., 2013). This migrating population could be the origin of around 69% of the RGLs in the SGZ of the young P15 DG, although their contribution at adult stages has not been estimated. Recently it has been shown that ventral and dorsal populations respond differentially to Shh signaling as *Sufu* deletion (acting in this context as a Shh signaling inhibitor) only impairs the proliferation of RGLs in the dorsal DG, but not in the ventral DG (Noguchi et al., 2019). This difference could be due to underlying molecular differences between RGLs and the surrounding cells residing in these regions. Nevertheless, it is still unclear how the caudal temporal population, or even the rostral septal RGL population, acquire the molecular and functional characteristics of adult RGLs.

## Developmental Origin of Quiescent Rgls

Even from early stages (E14.5), there are differences between the DG progenitors and those that will give rise to the hippocampus proper or the cortex. A subpopulation of GFAP expressing cells can be detected in the DGN, whereas in the adjacent dorsolateral neuroepithelium (cortical and hippocampal) RGCs do not express GFAP but Pax6 and BLBP. BLBP expression is acquired progressively in the GFAP expressing DG stem/progenitor cells from P1 to P14 (from 30 to 75% of total GFAP^+^ cells; Seki et al., 2014; Matsue et al., 2018). These results suggest that the properties of hippocampal granule stem/progenitor cells are rapidly altered from an embryonic to adult type soon after birth. But, what are those properties that define the adult RGLs? Is there a distinct population of specified RGLs or are the developmental RGCs that start behaving differently?

Perhaps one of the characteristics that distinguish adult RGLs most clearly from their embryonic counterparts is the acquisition of quiescence by which the adult RGLs remain for long periods out of the cell cycle, in G0. The state of G0 quiescence is shared with many somatic stem cells in other mature vertebrate tissues and is crucial to maintain tissue homeostasis and avoid stem cell exhaustion (Simons and Clevers, 2011; van Velthoven and Rando, 2019). In invertebrates such as *Drosophila*, quiescent neural stem cells can be arrested in either G0 or G2 (Otsuki and Brand, 2018).

**FIGURE 2:**
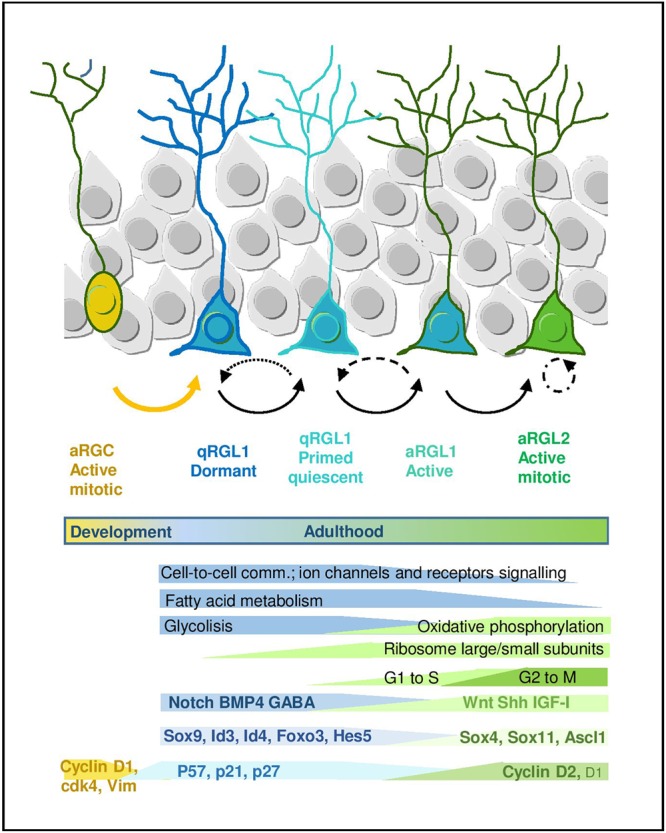
Quiescence and activation in RGL cells in the hippocampal neurogenic niche. Scheme representing the RGL cell stages during early lineage progression. Main molecular programs are highlighted (adapted from Llorens-Bobadilla et al., 2015 and Shin et al., 2015). It also includes molecular signatures discussed in the main text.

Taking a candidate gene approach, several groups have examined the role of cell cycle related genes in the regulation of RGL quiescence. There are some indications of cell cycle genes differentially involved in embryonic versus adult neurogenesis. Among them, we encounter the *CyclinD* genes, which are necessary for the mid-G1 cell cycle checkpoint. The three *CyclinDs* are differentially expressed in the brain regions during embryonic and postnatal stages (Glickstein et al., 2007). Surprisingly, *CyclinD2* but not *D1* mutation severely reduced proliferation of RGLs and progenitors in the SGZ from P7 onward causing almost a 10-folds less proliferation at P30 (Kowalczyk et al., 2004; Ansorg et al., 2012). These data indicate that postnatal neurogenesis is controlled by CyclinD2 together with at least one other D-type cyclin, and that the age at which DG neurogenesis becomes exclusively dependent on the expression of functional CyclinD2 lies between P14 and P28 (Ansorg et al., 2012; Figure 2). However, it is not clear if the importance of CyclinD2 is because it is enriched in adult RGLs or because CyclinD2 confers differences in cell cycle dynamics with respect to CyclinD1. In that sense, CyclinD1 can be only incompletely compensated for by knock in of *CyclinD2* into the *CyclinD1* locus, indicating non-redundant functions of these proteins (Carthon et al., 2005). Moreover, as *CyclinD2* mutants have also embryonic defects resulting in a reduced DG postnatally, conditionally removing *CyclinD2* from the adult niche is still required to establish if the defects in adult neurogenesis are due to a defect in the specification or maintenance of the adult RGL cell population during development.

Other cell cycle regulators that could be important in the establishment of the RGL pool are the cyclin-dependent kinase inhibitors. Among them, the Cip/Kip family which includes p21^Waf/Cip1^ (referred to as p21), p27^Kip1^ (referred to as p27), and p57^Kip2^ (referred to as p57). *p21* and *p27* deletion in mice during development results in increased progenitor proliferation in the hippocampus (Pechnick et al., 2008; Qiu et al., 2009). p27 is expressed in RGL cells in the adult DG and in the full *p27* mutant mice or in animals carrying a disruption in the cyclin-CDK interaction domain of p27 there is an increase in the proliferation of adult RGLs (Andreu et al., 2015). It is currently unclear if this leads to the loss of RGLs with time and/or if RGC proliferation is affected during DG development. Similarly, p57 is expressed in quiescent RGLs and its specific deletion in adult RGLs abrogates their quiescence (reduction in the number of RGLs that retain BrdU for long periods of time or BrdU-LRCs) and activates their proliferation (Furutachi et al., 2013). That leads to an increase in the number of new neurons, but subsequently at long-term (in 2 years old animals) it leads to excessive reduction of both RGLs and neurogenesis in the aged brain. It is not clear for how long the RGLs maintain high levels of proliferation in the p57 adult mutant before losing prematurely that potential in old animals. Furthermore, it has not been described what happens to hippocampal neurogenesis when p57 is removed during developmental stages.

Some clues about the possible relevance of p57 in establishing the pool of adult quiescent RGLs have emerged from the studies of the V-SVZ neurogenic niche in Nestin-cre/p57 mice (Furutachi et al., 2015). During embryonic ganglionic eminences development, the cell cycle of a subset of neural progenitor cells of the V-SVZ slows down, between E13.5 and E15.5, while other neural progenitors continue to divide rapidly (Fuentealba et al., 2015; Furutachi et al., 2015). A majority of RGLs in the young adult V-SVZ originate from these slowly dividing embryonic progenitors that are “set aside” and express high levels of p57. The conditional deletion of *p57* reduces the pool and proliferation of adult V-SVZ RGLs from E17.5 till at least P24 and also decreases the generation of new neurons. However, these studies do not exclude the possibility that some adult RGLs could derive from rapidly dividing embryonic progenitors, as adult RGLs are heterogeneous.

Unlike precursors for adult RGLs in the V-SVZ, recent results suggests that precursors for adult DG RGLs are not clearly “set-aside” dormant in quiescence during embryonic development, but instead seem to transition predominantly to a quiescent state postnatally. Hopx^+^ cells give rise to RGLs with quiescence properties (RGLs that retain BrdU after a 30 day pulse) from midgestation but the production clearly peaks during the first postnatal week (Berg et al., 2019). In parallel, another group has demonstrated that *Sufu* conditional deletion (which causes a reduction in Shh signaling) impairs the ability of RGLs to expand in the first postnatal week that results in the premature entry of RGLs into the quiescent state (Noguchi et al., 2019). These recent data suggest that Shh signaling activity must be continuously maintained to promote RGL expansion postnatally.

Apart from cell cycle inhibition, the quiescent state involves changes in cell adhesion molecules as well. A similar function to that of p57 in the establishment of the adult RGL pool has been suggested for vascular cell adhesion molecule-1 (VCAM) in the V-SVZ (Hu et al., 2017). Blocking VCAM function in adult SVZ, it was shown that VCAM1 is required for keeping adult RGLs in a quiescent state (Kokovay et al., 2012). Moreover, conditional deletion of VCAM1 in the embryonic brain caused RGCs premature differentiation during mid-gestation and reduced quiescence in slowly dividing RGCs with a reduction in the pool of postnatal and adult BrdU-LRCs (Hu et al., 2017). Thus, VCAM1 is required for the preservation of the embryonic RGCs into the adult stage. However, its possible function in the hippocampal niche has not been reported.

Altering the control of quiescence during development could provoke the exhaustion of the pool of active RGLs in mutant mice. For instance, loss of the phagocytosis factor Mfge8 during development promotes an increase in RGC proliferation at P15 and leads to the exhaustion of the neurogenic pool causing a decrease in RGL cell proliferation and neurogenesis by P30 (Zhou et al., 2018). Nevertheless, it is not clear if the depletion of RGLs in the adult brain is due to the premature overproduction or also to the fact that deletion of Mfge8 in the adult brain causes a change in cell fate specification and RGLs adopt an astrocytic fate (Zhou et al., 2018). It is also striking that in only 15 days (from P15 to P30) the pool of proliferating RGLs could be depleted. Moreover, the possible alterations in DG development and SGZ specification in Mfge8 mutants at earlier stages (before P15) have not been analyzed.

In summary, there are differences between the main adult neurogenic niches in the way and time the adult RGLs are generated. Thus, in the neuroepithelium of ganglionic eminences, neural precursors that give rise to adult RGLs (source of olfactory bulb interneurons) will be set aside at E13.5/15.5 from other precursors that will continue generating late born cortical interneurons and glial cells pre- and perinatally. In contrast, in the DG neuroepithelium (where development is protracted and cell lineage is very restricted), at around E15.5 subsets of precursors will continuously generate adult RGLs (source of only one type of neuron, the GN) with a clear peak in the first postnatal week, while the rest of DG precursors will generate also GN pre- and perinatally. These differences between adult niches could be probably related to early lineage specification and restriction during the development of their respective neuroepithelial progenitors, but the specific signals and mechanism involved in those early processes need to be revisited in the light of the generation of adult RGLs.

Nevertheless, the question of what exactly drives the acquisition of the adult RGL identity in the DG during early postnatal development remains open. In that sense, an in-depth analysis of the molecular program that controls the state of quiescence may eventually shed light on how quiescent RGLs become established. In addition, it is unclear if embryonic/postnatal quiescent RGLs are the same as adult quiescent RGLs.

## The Quiescence-Specific Gene Expression Program of Adult Rgls

Many efforts have been recently devoted to the identification of the molecular signature that defines the quiescent state of adult RGLs. The development of fluorescence-activated cell sorting protocols based on combinations of markers has allowed to prospectively isolate populations of quiescent and proliferating RGLs from the brain, facilitating the analysis of their transcriptomic fingerprint. The current cell sorting approaches rely on the use of: (1) a variety of cell surface epitopes to enrich in stem/progenitor cells, (2) transgenic animals expressing fluorescent proteins under the regulation of GFAP, Nestin and more recently LPA1 and Hopx for the hippocampus, and (3) fluorescent EGFR ligands to distinguish between active (EGFR^+^) vs. quiescent (EGFR^-^) RGLs (Capela and Temple, 2002; Beckervordersandforth et al., 2010; Daynac et al., 2013, 2015; Codega et al., 2014; Mich et al., 2014; Llorens-Bobadilla et al., 2015; Shin et al., 2015; Walker et al., 2016; Dulken et al., 2017; Morizur et al., 2018; Berg et al., 2019). Based on these isolation approaches, both genome-wide microarray, RNAseq and single-cell RNAseq transcriptomic datasets have been generated and some laboratories have even developed on-line tools that allow to explore the single-cell RNAseq data interactively^1,2^. In general, datasets obtained using different combinations of markers and strategies partly overlap and there is wide consonance in the functional interpretation of this valuable transcriptomic information (Figure 2).

A common theme uncovered in the multiple transcriptomic datasets through gene ontology and pathway analyses is the enrichment in genes related to lipid metabolism, glycolysis, cell signaling/communication and cell adhesion in the RGL quiescent state, both in the V-SVZ and DG. This profile is in sharp contrast to the enrichment in genes linked to the cell cycle, DNA/RNA metabolism, transcription and protein translation that characterizes the proliferative state (Llorens-Bobadilla et al., 2015; Shin et al., 2015; Morizur et al., 2018; Berg et al., 2019). In single-cell RNAseq studies, in which expression dynamics are reconstructed along a pseudo-time, the first mark for the exit from quiescence is the increase in the expression of genes that code for ribosomal proteins and that therefore participate in protein biosynthesis. Indeed, lentiviral overexpression of some ribosomal proteins is sufficient to induce proliferation of adult DG RGLs (Mukherjee et al., 2016). The upregulation of ribosomal genes is followed by a shift in energy metabolism genes from glycolysis to mitochondrial oxidative phosphorylation (Llorens-Bobadilla et al., 2015; Shin et al., 2015). Fatty acid metabolism has also emerged as a crucial regulator of RGL activity in the DG. *De novo* lipogenesis is key for proliferation (Knobloch et al., 2013) while lipid breakdown through FAO in the mitochondria is most prominently required in the quiescent state (although FAO is also used by proliferating cells). Whole proteome analysis of active vs. quiescent stem/progenitor cell cultures corroborates the transcriptomic data, evidencing an increase in FAO in quiescence, and metabolic experiments show that FAO is required during quiescence for energy production and as an alternative carbon source (Knobloch et al., 2017). Recent data also suggest similar shifts in lipid metabolism, cell cycle and translation in DG progenitors coinciding with the transition from postnatal RGCs to adult quiescent RGLs (Berg et al., 2019).

Transcriptomic data comparing quiescent and activated V-SVZ RGLs have also uncovered differences in the protein homeostasis network (Leeman et al., 2018). Activated RGLs mostly rely on proteasomal degradation for protein quality control while quiescent RGLs accumulate protein aggregates in lysosomes, a phenomenon that is exacerbated during aging. *In vitro*, stimulating the lysosome-autophagy pathway clears the protein aggregates and enhances RGL activation in response to growth factors. Generally speaking, metabolic changes are required to meet the increased biosynthetic demands of actively proliferating cells and are thus observed during the exit from quiescence of many cell types, including adult somatic stem and non-stem cells (García-Prat et al., 2017; Chapman and Chi, 2018). It is currently unclear if the aforementioned metabolic pathways (FAO, glycolysis, protein homeostasis) are merely adaptive responses to cell cycle withdrawal of quiescent cells or perhaps also drivers of RGL transformation *in vivo* during postnatal development.

In addition to the metabolic genes, transcription factors (TFs) are also differentially regulated in the quiescence-to-proliferation transition of RGLs, both in the DG (Shin et al., 2015) and the V-SVZ (Llorens-Bobadilla et al., 2015; Morizur et al., 2018). Some of the TFs (or their paralogs) are involved in embryonic neurogenesis and/or in the regulation of somatic stem cells in other non-neural adult niches. This suggests the existence of stage-specific genetic programs that would be controlled in a coordinated manner by those TF codes. The systematic analysis of the Shin et al. (2015) dataset, for instance, revealed around 80 TFs up and downregulated during the initial stages of adult hippocampal neurogenesis. As expected, TFs and transcription regulators such as *Sox9, Id4*, or *Id3* appear associated with the quiescent state in the hippocampus, while SoxC TFs (*Sox4* and *Sox11*) raise during activation, yet more than half of the regulated TFs identified in the dataset are still largely unexplored in the context of RGL quiescence (Shin et al., 2015). Similarly, in the V-SVZ *Sox9, Id2*, and *Id3* are associated with the quiescent state while *Ascl1, Sox4*, and *Sox11* are enriched in the active sate (Llorens-Bobadilla et al., 2015). Despite the indisputable relevance of these mRNA profiles, we should keep in mind that some of the TFs involved in the activation of RGLs are finely regulated post-translationally, as it is the case for Ascl1, a major player in shaping the transcriptomal landscape of active RGL and IPCs (Andersen et al., 2014; Llorens-Bobadilla et al., 2015). The E3-ubiquitin ligase Huwe1 destabilizes the Ascl1 protein in proliferating DG RGLs, preventing the accumulation of cyclinDs and returning the cells to the quiescent state (Urban et al., 2016). Supporting Ascl1 pivotal role of, it has been shown that Ascl1 oscillations, which in turn depend on Hes1 oscillations, regulate RGL activation, while high Hes1 expression and the resulting Ascl1 suppression promote DG RGL quiescence (Sueda et al., 2019).

Another characteristic of the quiescent state of RGLs is the upregulation of genes that code for membrane proteins involved in intercellular communication, cell adhesion and transport. In the DG for instance, among the top 1,000 quiescence-enriched genes downregulated during activation, 51% encode proteins associated with the membrane (Shin et al., 2015). Quiescent RGLs overexpress at the mRNA level both receptors of multiple relevant pathways in the SGZ niche (such as Dll/Notch, BMP, Insulin, FGF, or neurotrophins) and receptors of the neurotransmitters GABA, glutamate and calcium channels, suggesting that quiescent RGLs are probably more sensitive to extrinsic stimuli than their committed progeny (Shin et al., 2015). Cell membrane genes are also markedly increased in DG progenitors over development, pointing to a switch from an intrinsic mode of regulation in RGCs to a niche-dependent mode in adult RGLs (Berg et al., 2019). However, we should not forget that this scheme is based on the extrapolation of the transcriptomic information to the membrane proteome and to its potential signaling activity, not on direct signaling measurements. Given the enormous post-transcriptional regulation and recycling by internalization suffered by membrane receptors, functional correlations are not guaranteed. An example of the mRNA/membrane protein discrepancy has already been reported for members of the transmembrane syndecan family heparan sulfate proteoglycans in quiescent RGLs of the V-SVZ (Morizur et al., 2018).

In addition to the membrane receptors, several cell adhesion proteins, including neural cell adhesion molecules and cadherins/protocadherins are overexpressed in quiescent RGLs (Morizur et al., 2018). This further highlights the key role of cell adhesion and niche interaction in the modulation of RGL activity, as described previously for VCAM1 (Kokovay et al., 2012) or N-cadherin in V-SVZ RGLs (Porlan et al., 2014).

In summary, the transcriptomic data have allowed to study the molecular signature of quiescent and active RGLs, and based on the available information, new concepts are already emerging. The transcriptomic data have been partly endorsed by functional assays employing previously established *in vitro* quiescence protocols (Mira et al., 2010; Martynoga et al., 2013) and to a lesser extent, employing prospectively isolated cells or transgenic mouse models. Together, the transcriptomic, proteomic and functional data point to profound bioenergetic and cell signaling differences between quiescent RGLs and their active counterparts and highlight the potential of metabolic pathways as direct regulators of RGL transitions, as shown in other adult stem cell populations (García-Prat et al., 2017). Future mechanistic studies will be fundamental to gain insight on their putative role on the acquisition of the RGL identity and quiescence during development.

## Insights Into the Developmental Origin of Hippocampal Rgl Cells Through Single-Cell Transcriptomics

This question has been only directly addressed by the Linnarsson lab (Hochgerner et al., 2018). They performed a large-scale single-cell RNA-seq analysis of DG cell types throughout development and into adulthood, including RGCs and adult RGLs, but also neuroblasts, GN, astrocytes and other cell types (none of the latter analyzed in Shin et al., 2015). The power of the study relies on the vast number of cells analyzed (>24,185 cells), the unbiased sampling, the inclusion of perinatal, juvenile, and adult DG tissue, and the use of two complementary platforms to minimize batch effects. The data strongly support that adult neurogenesis from development to adulthood, proceeds through a set of defined cellular states and transitions. Thus, while IPCs, neuroblasts and immature neurons express sequentially a conserved neurogenic TF cascade (Sugiyama et al., 2013) and are nearly indistinguishable across time, RGCs/RGLs, by contrast, display great shifts in their molecular profile as development proceeds (see below). Moreover, RGLs do not express cell-cycle genes such as *Top2a* or *Cdk1*, basically because they mostly identify the adult quiescent RGLs. The small population of actively proliferating RGLs probably cluster with IPCs, but can be distinguished because dividing RGLs have not entered the neurogenic program, while quiescent RGLs appear as a defined population disconnected from the main neurogenic trajectory that starts from the IPCs (yet this could be an artifact of visualizing the cells using t-distributed stochastic neighbor embedding (t-SNE) plot, see Kalamakis et al., 2019). RGLs are clearly distinct from astrocytes, expressing for instance TFs such as *Sox4* and *Ascl1*, or *Thrsp* (SPOT14), a key lipid metabolism regulator that when knocked down leads to a shift in quiescent RGLs toward more proliferative progenitors (Knobloch et al., 2013). According to the Linnarsson study, the key event in DG neurogenesis would be the cellular decisions occurring at the time of RGL activation, after which cells proceed to the IPC state, upregulating neurogenic TFs and engaging irreversibly in a conserved neuronal fate program, at least under non-pathological conditions.

But perhaps the most interesting finding of the Linnarsson lab is that related to the developmental origin of the adult RGL population (Hochgerner et al., 2018). The comparison of cells across time, from E16.5 to adulthood (P132) allowed them to suggest that the transcriptome of RGCs is profoundly affected as they transition to RGLs. Perinatal RGCs (E16.5, P0, and P5) are distinguishable from juvenile and adult RGLs, in that they express *Vim*, display higher expression of *Sox4* and *Sox11* and lower of *Notch2* and *Padi2*. Moreover, in relation to cell cycle changes, embryonic RGCs show comparatively higher levels of *Cdk4* than juvenile RGL, whereas RGLs express higher levels of *Cdk1* than RGCs. It remains to be analyzed if those changes in Cyclin-dependent kinase could be related to the temporal differences in Cyclins that we discussed above. Thus, they find that although postnatal and adult neurogenesis in the hippocampus is fundamentally similar, there is an early postnatal transformation of RG from embryonic progenitors (RGCs) to adult quiescent stem cells (qRGLs, Figure 2).

In this same line, the Song lab compared, by RNAseq, pools of mixed neural progenitors from the Hopx-creERT mouse hippocampus at different stages (E15.5 and P3) and from the adult DG at P45. However, a caveat of the study is that it relies on a gene, Hopx, that is dynamically expressed at early stages in progenitors for both the DG and CA and at adult stages in quiescent RGLs but is absent from adult IPCs. They identified a shared signature of 1,306 genes among all Hopx-creERT labeled progenitors, supporting their developmental relationship. GO analysis revealed consistent changes over time in several gene sets, an observation interpreted by the authors as a gradual and continuous transformation of progenitor cells over the course of DG development. Nevertheless, as in the Linnarsson’s model, the most abrupt progenitor DG transition at the molecular level occurs postnatally. Marked gene expression differences are detected between the early postnatal and adult progenitors, including for instance the downregulation of the cell cycle genes encoding CyclinD1 and D2 and the upregulation of the cell cycle inhibitor p21. However, these data could reflect just the difference between the pool of embryonic Hopx-creERT neural precursor (the majority of which will be cycling) with respect to the exclusively quiescent RGL population labeled in the adult Hopx-creERT line.

In summary, all these transcriptomic data are in accordance with previous histological observations describing the temporal heterogeneity of RGCs and RGLs in the DG at the level of marker expression, and already showing that, structurally, the “adult” configuration of SGZ niche gets established between the first and second postnatal weeks, before individuals reach “adulthood” (Nicola et al., 2015).

Linnarsson and co-workers also described a fast maturation of GNs and mossy cells around the third postnatal week. It has been previously suggested that the establishment of the commissural fiber tract of the DG around P15 (Fricke and Cowan, 1977; Ribak et al., 1985) might have an impact on DG development (Nicola et al., 2015). RGL quiescence is also regulated by the input from contra- and ipsi-lateral mossy cells and by long-range GABAergic projections from the medial septum (Bao et al., 2017; Yeh et al., 2018). For that reason, it is conceivable that postnatal changes in connectivity contribute to the establishment of the quiescent RGL reservoir and the formation of the adult DG niche, as previously proposed (Nicola et al., 2015). The single-cell RNAseq resource generated by the Linnarsson lab may allow to further explore at the molecular level which genes are key players in the postnatal transition from RGCs to RGLs^3^.

## Conclusion

We have witnessed a breakthrough in transcriptomic and proteomic analyses of the quiescence state of adult RGLs and in the signals and TFs involved in the transition from quiescence to activation in the adult brain. Moreover, recent data have uncovered a common origin from Hopx-expressing progenitors for embryonic RGCs, postnatal and adult RGLs in the hippocampal niche, although additional embryonic origins cannot be ruled out. Further mechanistic studies are indeed still warranted to define how exactly are quiescent RGLs specified during the early postnatal period and what is the precise role, if any, exerted by the players we discussed above in the process. Another important question is how the differences in gene expression found between embryonic RGCs and adult RGLs determine their cycling, lineage and cell-to-cell communication behavior. With all the available datasets, these fundamental aims are within reach. Overall, the understanding of the generation of new neurons in the adult brain through the control of the establishment and regulation of the quiescent RGL reservoir is of paramount importance in order to harness quiescent RGLs into neurogenic production in pathological and aging situations.

## Data Availability

No datasets were generated or analyzed for this study.

## Author Contributions

AM and HM conceived the structure and content and wrote the manuscript. AM produced the figures.

## Conflict of Interest Statement

The authors declare that the research was conducted in the absence of any commercial or financial relationships that could be construed as a potential conflict of interest.

## Footnotes

1 https://martin-villalba-lab.shinyapps.io/scRNAseq_CSC2015/

2 https://linnarssonlab.org/dentate/

3 https://linnarssonlab.org/dentate/

## Abbreviations

BLBP: brain lipid binding protein
BMP: bone morfogenetic protein
DG: dentate gyrus
DGN: dentate gyrus neuroepithelium
EGFR: epidermal growth factor receptor
FAO: fatty acid oxidation
FGF: fibroblast growth factor
GABA: gamma-aminobutyric acid
GFAP: glial fibrillary acidic protein
GN: granule neuron
IPC: intermediate progenitor cell
LPA1: lysophosphatidic acid receptor 1
RGC: radial glial cell
RGL: radial glia-like cell
SGZ: subgranular zone
VCAM1: vascular cell adhesion molecule 1
V-SVZ: ventricular-subventricular zone
